# Associations between AGT M235T Polymorphism and Cancer: An Updated Meta-Analysis

**DOI:** 10.1155/2022/7862709

**Published:** 2022-03-04

**Authors:** Junyan Kou, Jing Huang

**Affiliations:** Department of Integration of Western and Traditional Chinese Medicine, Hangzhou Cancer Hospital, Hangzhou 310000, China

## Abstract

We assessed the relationship between AGT gene M235T polymorphism and the susceptibility to cancer by performing an updated meta-analysis. This study retrospectively searched related articles in the electronic databases. Afterwards, we determined combined odds ratios (ORs) and related 95% confidence intervals (CIs) by the fixed- or random-effects model. The present meta-analysis enrolled altogether 9 articles. On the whole, the relationship between AGT M235T polymorphism and the cancer risk was not significant among the entire population (TT vs. MM: OR = 1.28, 95%CI = 0.80 − 2.04; TM vs. MM: OR = 0.90, 95%CI = 0.53 − 1.52; recessive model: OR = 1.13, 95%CI = 0.83 − 1.52; dominant model: OR = 0.93, 95%CI = 0.55 − 1.57). Subgroup analysis by ethnicity, cancer type, and study quality for the relationship between the AGT M235T polymorphism and cancer risk showed no significant association. According to findings in the present meta-analysis, AGT M235T polymorphism may not be related to cancer susceptibility.

## 1. Introduction

Cancer greatly affects the global economy and public health. According to statistics, 14 million cancer patients are diagnosed in 2012, and the cancer morbidity is predicted to increase to nearly 22 million in 2030 [1]. At present, the cancer pathogenic mechanism remains largely unclear, and cancer is reported as a complicated condition induced by numerous factors, such as genetic factors, smoking, excessive drinking, chemical dyes, high-calorie diet, or their combination [2]. Typically, genetic factors are recognized to exert vital parts in cancer risk, with numerous cancer pathogenesis-related genes being identified as the cancer risk genes [3].

Renin-angiotensin system (RAS) is the hormone signaling pathway, which has been suggested to modulate blood pressure (BP) and cardiovascular homeostasis. Besides, RAS within local tissues is possibly associated with cancer genesis and progression [4]. In brief, renin can release 10 amino acids (aa) in angiotensinogen (AGT) for forming Ang I as well as the great protein (des (Ang I) AGT). Both des (Ang I) AGT and AGT have been recognized as the noninhibitory serpins inhibiting new blood vessel formation [5]. Then, ACE can eliminate the above 2 aa from Ang I for generating Ang II. Notably, Ang II represents a major RAS active peptide that can promote cell proliferation and new blood vessel formation via angiotensin II type 1 receptor (AGTR1) [6].

AGT gene is 12,068 bp in length and located on chromosome 1q42.2, and there are 4 introns and 5 exons in the gene coding region. Mutations in the AGT gene mostly result from thymine nucleotide (T) substitution by cytosine nucleotide (C) at the +704 position in exon 2. Therefore, the codon 235-encoded methionine (Met) is replaced by threonine (Thr) (also referred to as M235T), and 2 alleles are formed, including 235T (variant type) and 235M (wild type). Altogether, 3 genotypes are detected among the population, which are homozygous 235TT and 235MM, as well as heterozygous 235M [7]. As discovered by Paillard and colleagues, AGT-235 T allele elevated the plasma AGT content [8], which induced smooth muscle proliferation and contraction of small arteries, lipid deposition, and hypertrophy of vascular smooth muscle cells (VSMCs); increased norepinephrine production; and excited the sympathetic nervous system.

According to previous meta-analysis, the M235T variant in the AGT gene is not related to the susceptibility to cancer [9]. But that meta-analysis only involves a small sample size and does not take into account some latest studies. The aim of the present study was to compile case-control research and updated meta-analyses to explore the association between AGT M235T polymorphism and susceptibility for cancer, so as to more accurately assess the cancer risk.

## 2. Materials and Methods

### 2.1. Study Search Strategy

This meta-analysis was carried out independently in line with guidelines of the preferred reporting items for systematic reviews and meta-analyses (PRISMA) [10]. Electronic databases of PubMed, Web of Science, and CNKI were searched for identifying articles that examined the association of AGT M235T polymorphism with cancer risk from inception to March 1^st^, 2021, using the keywords below: (“Angiotensinogen” OR “AGT”) and (cancer OR “tumor” OR “carcinoma”) and (“polymorphism” OR “mutation” OR “genotype” OR “allele” OR “variation” OR “variant”). At the same time, the reference lists in related studies were manually searched to avoid omitting any eligible study. No language restriction was applied in literature retrieval.

### 2.2. Inclusion Criteria

Eligible researches were enrolled according to the inclusion criteria: (1) studies that assessed the relationship of AGT M235T polymorphism with cancer risk, (2) case-control studies, and (3) those with available genotyping information. The following was the exclusion criteria: (1) articles not related to cancer, (2) reviews, (3) articles with no available data, and (4) duplicates.

### 2.3. Data Extraction

Two investigators reviewed the related articles and collected data, and any disagreement between them was settled by the opinion of a third reviewer. The following information was collected, including first author, region, publication year, case and control numbers, and case and control genotype frequencies, together with Hardy–Weinberg equilibrium (HWE) of the control group.

### 2.4. Quality Evaluation

According to Table 1, the quality assessment rules were used for quality evaluation of the articles [11]. In brief, study quality was evaluated based on control source, sample size, case representativeness, diagnosis of cancer, genotyping quality evaluation, and HWE, and the overall score was between 0 and 15. Studies that had a score ≥ 10 were deemed as high quality, while those that had a score < 10 as “low quality”.

### 2.5. Statistical Methods

Meta-analysis was carried out by adopting STATA12.0. ORs together with related 95% CIs were employed for evaluating correlation of AGT M235T polymorphism with cancer susceptibility under different comparisons, including heterozygote (TM vs. MM), homozygote (TT vs. MM), recessive model (TT vs. MM+TM), and dominant model (TT+TM vs. MM) between groups. Moreover, the *χ*^2^ test was utilized for determining HWE regarding the distribution of genotype among all the enrolled researches. Heterogeneity was analyzed by *I*^2^ statistic, and *I*^2^ > 50% suggested heterogeneity. Subgroup analyses according to cancer type, ethnicity, and quality scores were also conducted. Afterwards, this study also conducted sensitivity analysis through eliminating a single study each time, showing suspect on one study of excessive sensitivity because the omission of this specific study yielded to the estimation beyond the 95% CI of the pooled analysis. Finally, we evaluated Begg's funnel plot for possible publication bias.

## 3. Results

### 3.1. Study Features

**Figure 1:**
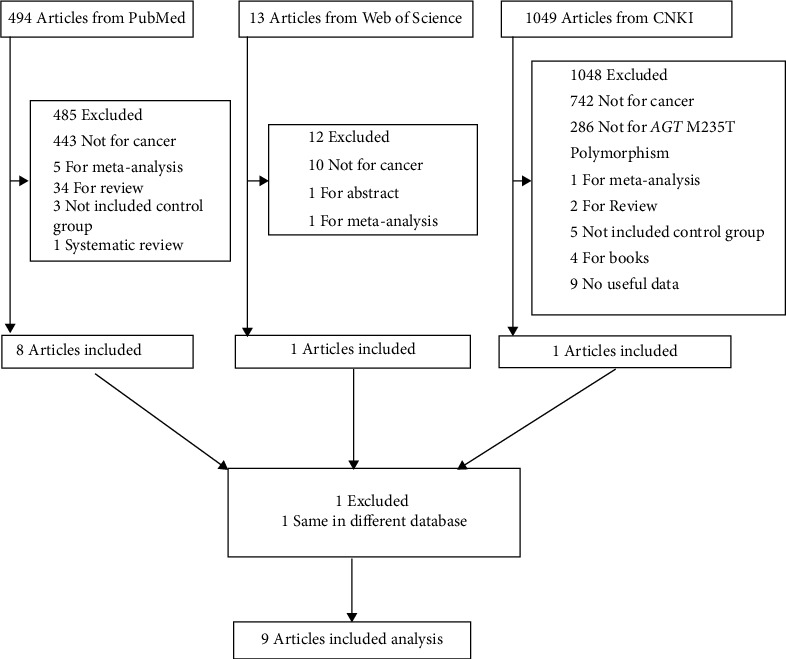
The flow diagram of included/excluded studies.

Altogether, 699 related studies were searched; at last, nine of them were included into the present meta-analysis according to the predetermined study inclusion and exclusion criteria [12–20]. All our collected articles were published from 2007 to 2020.  Figure 1 shows the study screening flow chart. In brief, the HWE test was carried out in the nine studies to examine the distribution of genotype in the control group. As a result, all studies did not deviate from the HWE, with the exception of Papaggelopoulos et al. and Pringle et al. All the enrolled studies had the quality score > 10 points, with the exception of Papaggelopoulos et al., indicating that these studies had high study quality. With regard to cancer type, 3 studies were on digestive cancer and 3 were on breast cancer (BC) to examine AGT M235T polymorphism.  Table 2 displays the study features and methodological quality.

### 3.2. Meta-Analysis

**Figure 2:**
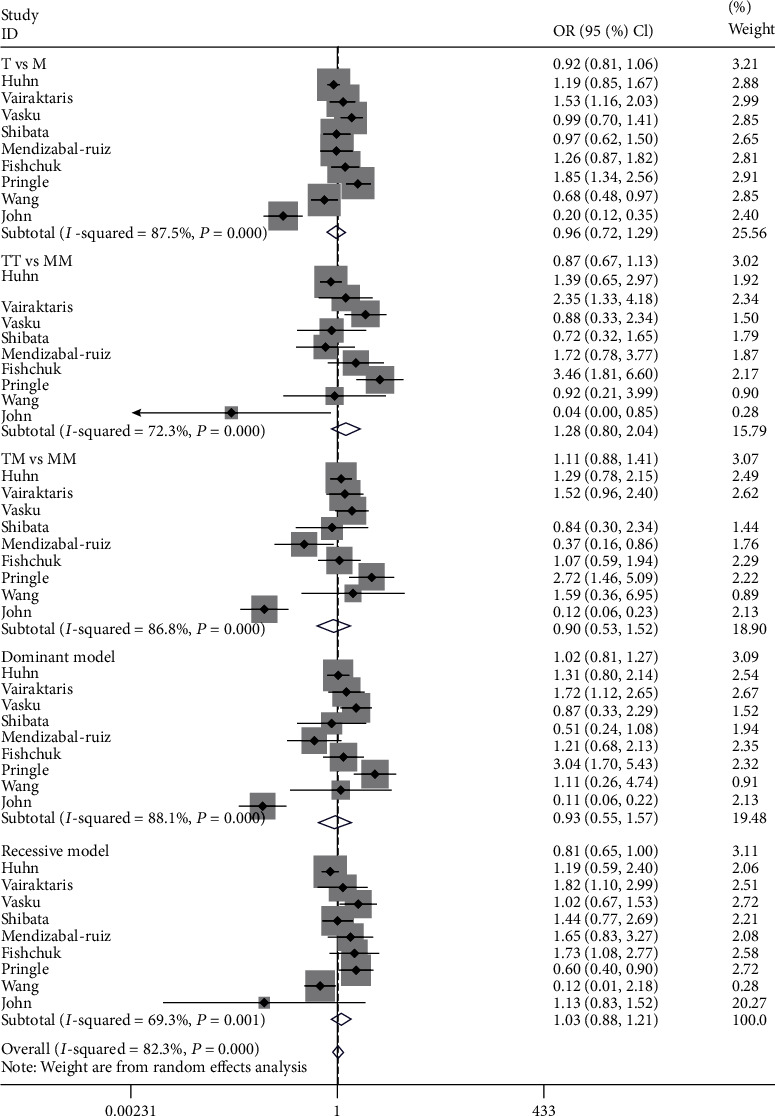
Forest plot for meta-analysis of the association between the M235T polymorphism and cancer risk with TT vs. MM.

Table 3 lists the major findings from this meta-analysis and the heterogeneity. On the whole, AGT M235T polymorphism did not show significant relationship with cancer under each genetic model (Figure 2, TT vs. MM: OR = 1.28, 95%CI = 0.80 − 2.04; TM vs. MM: OR = 0.90, 95%CI = 0.53 − 1.52; recessive model: OR = 1.13, 95%CI = 0.83 − 1.52; dominant model: OR = 0.93, 95%CI = 0.55 − 1.57).

As revealed by subgroup analysis stratified by ethnicity, AGT M235T polymorphism did not show significant relationship with cancer susceptibility in the Caucasian or the Asian population. Meanwhile, subgroup analysis stratified by quality score suggested that AGT M235T polymorphism did not show significant relationship with cancer susceptibility. According to subgroup analysis based on cancer type, no significant association was found in digestive cancer and breast cancer.

### 3.3. Sensitivity Analysis

**Figure 3:**
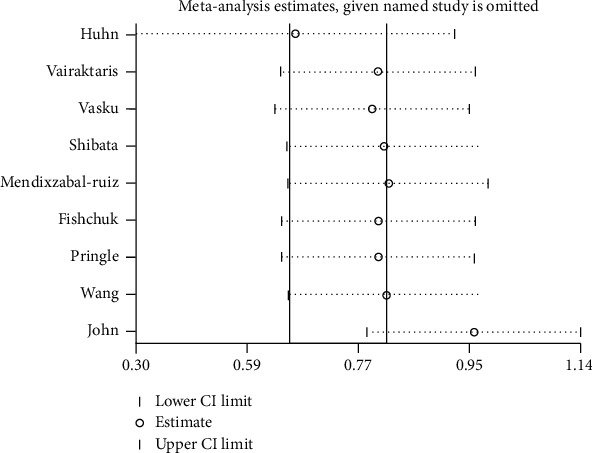
Sensitivity analysis of the association between the M235T polymorphism and cancer risk.

When each individual study was eliminated, the pooled results were not changed, which indicated statistical significance of our results (Figure 3).

### 3.4. Publication Bias

**Figure 4:**
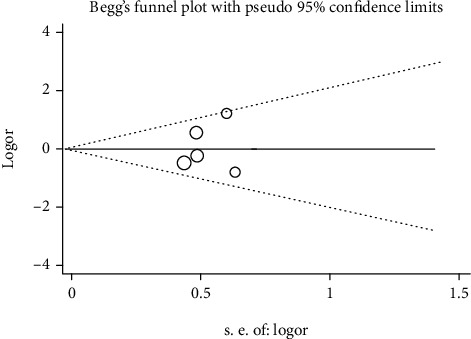
Begg's funnel plot analysis to detect potential publication bias for M235T polymorphism.

We drew the Begg funnel plot for assessing the possible publication bias among the enrolled articles. No evident asymmetry was observed in the funnel plot (Figure 4, Begg's test *p* = 1.000).

## 4. Discussion

At present, cancer is recognized to be a major cause leading to mortality in the world. It has brought severe social and economic burdens on the health-care system across diverse countries; what is worse, it has deteriorated the patient life quality [21]. Regardless of the progresses made in cancer treatment, cancer prognosis is still poor. Cancer represents a kind of multifactorial disorder. As reported in some studies, the interaction between polymorphisms and environmental factors exerts a vital part in cancer genesis. More and more studies find that RAS affects cell proliferation, inflammation, apoptosis, and tissue angiogenesis. However, most existing case-control studies are conducted to examine the relationship of AGT M235T polymorphism with the susceptibility to cancer. Nonetheless, no consistent results are obtained. Previous meta-analysis demonstrated that the AGT M235T variant was not associated with risk of all cancer or various cancers [9]. However, the reference 10 in the article is aimed at AGT-20 A/C polymorphism, but not M235T [22]. Previous meta-analysis included references 10 for meta-analysis, and the final result is inaccurate. In recent years, four relevant articles have been published. This updated meta-analysis was carried out for assessing the relationship of AGT M235T polymorphism with the susceptibility to cancer.

Our findings suggest that this polymorphism was not related to a higher susceptibility to cancer. As revealed by subgroup analysis stratified by race, AGT M235T polymorphism was not associated with cancer risk among the Asian and Caucasian populations. Discrepancies between studies could be possibly due to a different role of this polymorphism in different cell types or tissues. When stratified by cancer type, AGT M235T polymorphism was not associated with digestive cancer and breast cancer. Probably, the non-HWE studies were associated with the potential selection bias or genotyping error, thereby leading to misleading findings. Furthermore, subgroup analysis was also carried out to remove studies with deviated genotype distribution from the HWE in the control group, and no altered result was detected, indicating the result of meta-analysis was statistically significant.

These findings suggest that the risk of cancer may be not related to AGT M235T polymorphism or that so far, research has been insufficient to identify such association. There are several potential explanations for the negative results. Firstly, the connection of AGT M235T polymorphism with cancer risk may be influenced by genetic relationships. AGT A-20C polymorphism is reported previously to predict a higher susceptibility to cancer [22]. There is a linkage disequilibrium of A-20C with M235T polymorphism sites of the AGT gene [23]. These two polymorphisms may cooperate with each other to add the risk of disease. Secondly, such negative findings may also be related to the great heterogeneity among the included studies into the current analysis. Heterogeneity can be derived from any variation in terms of genetic constitution and/or environmental trait among different populations, as well as the different sample selection criteria (such as age, sex, and diagnostic criteria) and the different study designs [24]. Thirdly, a number of environmental factors, like smoking, are related to the occurrence of cancer. Therefore, these variables must be examined in subsequent articles, regardless of the difficulties in assessing environmental exposures and study design [2].

Certain limitations must be noted in the present meta-analysis. Firstly, raw data from the enrolled articles were lacking, which restricted our ability to better evaluate the associations between genes and between genes and the environment. Secondly, each of the enrolled articles was of retrospective nature, which might inevitably lead to subject selection bias, finally impacting our result reliability. Thirdly, the present meta-analysis just enrolled the published articles, while the related unpublished articles were not enrolled, which might cause a potential publication bias.

To sum up, our meta-analysis reveals that AGT M235T polymorphism is not related to cancer risk. A preprint has previously been published [25]. More large-scaled case-control studies should be conducted to explore the potential relationships between genes and between genes and the environment with cancer incidence.

## Figures and Tables

**Table 1:** Scale for quality assessment.

Criteria	Score
Source of cases	
Selected from population or cancer registry	3
Selected from hospital	2
Selected from pathology archives, but without description	1
Not described	0
Source of controls	
Population-based	3
Blood donors or volunteers	2
Hospital-based (cancer-free patients)	1
Not described	0
Specimens of cases determining genotypes	
White blood cells or normal tissues	3
Tumor tissues or exfoliated cells of tissue	0
Hardy-Weinberg equilibrium in controls	
Hardy-Weinberg equilibrium	3
Hardy-Weinberg disequilibrium	0
Total sample size	
≥1000	3
≥500 but <1000	2
≥200 but <500	1
>0 but <200	0

**Table 2:** Characteristics of the included studies of AGT M235T polymorphism.

Study included	Year	Cancer type	Race	Cases/controls	Allele for cases		Allele for controls		Genotypes for cases			Genotypes for controls			HWE test	Quality scores
					T	M	T	M	TT	TM	MM	TT	TM	MM		
Vairaktaris et al.	2008	Oral cancer	Caucasian	163/124	133	193	91	157	23	87	53	15	61	48	0.51	10
Vasku et al.	2009	Colorectal cancer	Caucasian	102/101	200	200	158	242	50	100	50	31	96	73	0.95	10
Shibata et al.	2011	Gastric cancer	Asian	206/210	337	75	344	76	140	57	9	142	60	8	0.60	10
Mendizábal-Ruiz et al.	2011	Breast cancer	Mixed	50/224	59	41	268	180	21	17	12	75	118	31	0.15	10
Huhn et al.	2012	Colorectal cancer	Caucasian	962/760	980	944	804	716	244	492	226	225	354	181	0.07	12
Fishchuk and Gorovenko	2013	Breast cancer	Caucasian	131/102	124	138	85	119	29	66	36	15	55	32	0.27	10
Pringle et al.	2016	Endometrial cancer	Caucasian	183/133	239	127	134	132	78	83	22	40	54	39	0.03	7
Wang et al.	2016	Mixed	Asian	104/1178	164	44	1991	365	62	40	2	838	315	25	0.47	12
Papaggelopoulos et al.	2019	Basal cell carcinoma	Caucasian	91/99	20	162	75	123	0	20	71	4	67	28	0.00	6

HWE: Hardy-Weinberg equilibrium.

**Table 3:** Summary ORs and 95% CI of AGT M235T polymorphism with cancer risk.

Variables	*N* ^a^	T vs. M		TT vs. MM		TM vs. MM		Dominant model		Recessive model	
		OR (95% CI)	Model	OR (95% CI)	Model	OR (95% CI)	Model	OR (95% CI)	Model	OR (95% CI)	Model
Total	9	0.96 (0.72-1.29)	R	1.28 (0.80-2.04)	R	0.90 (0.53-1.52)	R	0.93 (0.55-1.57)	R	1.13 (0.83-1.52)	R
*Race*											
Asian	2	0.82 (0.57-1.19)	R	0.89 (0.40-2.01)	F	1.06 (0.47-2.39)	F	0.94 (0.42-2.08)	F	0.78 (0.46-1.31)	R
Caucasian	6	1.00 (0.67-1.51)	R	1.50 (0.81-2.80)	R	0.97 (0.52-1.83)	R	1.01 (0.53-1.93)	R	1.27 (0.83-1.97)	R
*Cancer type*											
Breast cancer	2	1.13 (0.85-1.50)	F	1.13 (0.48-2.63)	R	0.66 (0.23-1.84)	R	0.81 (0.35-1.89)	R	1.53 (0.97-2.43)	F
Digestive cancer	4	1.12 (0.87-1.44)	R	1.26 (0.73-2.16)	R	1.19 (0.98-1.44)	F	1.15 (0.96-1.38)	F	1.10 (0.76-1.59)	R
*Quality*											
High	7	1.05 (0.87-1.27)	R	1.19 (0.82-1.74)	R	1.13 (0.95-1.35)	F	1.11 (0.94-1.32)	F	1.08 (0.80-1.45)	R
Low	2	0.62 (0.07-5.48)	R	0.49 (0.01-41.59) R		0.57 (0.03-12.34)	R	0.58 (0.02-14.93)	R	0.66 (0.05-8.64)	R

^a^Number of comparisons; OR: odds ratio; CI: confidence interval; R: random model; F: fixed model.
